# ﻿Geometric morphometric characterization of the Balkan alpine grasshopper genus *Oropodisma* Uvarov, 1942 (Orthoptera, Acrididae, Melanoplinae), with description of two new species

**DOI:** 10.3897/zookeys.1240.150223

**Published:** 2025-06-05

**Authors:** Marina Trillo, Joaquín Ortego

**Affiliations:** 1 Department of Ecology and Evolution, Estación Biológica de Doñana, EBD-CSIC, Seville, Spain Estación Biológica de Doñana Seville Spain

**Keywords:** Alpine ecosystems, Balkan Peninsula, geometric morphometrics, Mediterranean mountains, microendemism, speciation

## Abstract

In this study, we employ a geometric morphometric approach to quantify morphological differences among taxa of the genus *Oropodisma* Uvarov,1942, a complex of alpine grasshoppers comprising several narrow endemic species distributed across different mountain ranges of the Balkan Peninsula. The genus was described by Uvarov in 1942 and currently includes 10 recognized species, primarily distinguished by subtle differences in the shape of the male phallus apex and furculae. Some populations from central Greece exhibit an uncertain taxonomic status, suggesting the possible existence of undescribed species. Quantitative morphological comparisons with specimens collected from the type localities of all currently recognized taxa revealed the presence of two new species in the Pindus Range, Greece: *O.tzoumerkae***sp. nov.** from Mount Tzoumerka and *O.agrafae***sp. nov.** from Mount Agrafa. Geometric morphometric analyses indicate that *O.tymphrestosi* and *O.willemsei*, which occur in adjacent mountain ranges from central Greece, do not exhibit statistically significant differences in the shapes of either the phallus apex or the furculae. This finding raises doubts about their current taxonomic status but may also reflect the limitations of geometric morphometric approaches in capturing subtle differences in the complex structures that characterize male genitalia. Overall, our analyses highlight the value of quantitative approaches in formally reassessing the taxonomic status of species complexes. The description of two new and geographically restricted species within *Oropodisma* further emphasizes the role of Mediterranean mountains as key centers of microgeographic speciation for alpine biotas.

## ﻿Introduction

Mediterranean mountains harbor high levels of local microendemism, with many taxa of great conservation value exclusively distributed on a single or a few nearby mountain tops (e.g. Peñas et al. 2005; Jiménez-Mejías et al. 2015; Ortego and Knowles 2022). This is the case of the grasshopper genus *Oropodisma* Uvarov, 1942 (Orthoptera, Acrididae, Melanoplinae), which includes several narrow-endemic species with fragmented populations at high elevations in different mountain ranges of the Balkan Peninsula (Cigliano et al. 2025). The genus *Oropodisma* was described in 1942 by the prominent acridologist Boris Petrovitch Uvarov based on specimens of *O.parnassica* (Scudder, 1897) collected on Mount Parnassos, Greece (Uvarov 1942). *Oropodismaparnassica* had already been described by Samuel Hubbard Scudder in 1897 from specimens collected by Hofrath Brunner von Wattenwyl in the same mountain range. However, this taxon was originally assigned to the genus *Podisma* Berthold, 1827 (Scudder 1897), later reassigned to *Cophopodisma* Dovnar-Zapolskij, 1932 (Dovnar-Zapolskij 1932), and ultimately placed in *Oropodisma* (Uvarov 1942). All other currently recognized species of *Oropodisma* were subsequently described within this genus (Uvarov 1942; Ramme 1951; Willemse 1971, 1972; La Greca and Messina 1977; Willemse 1979), which now comprises a total of 10 species that have never undergone further taxonomic revision (Cigliano et al. 2025). All species exhibit a very similar external appearance and were primarily described based on subtle differences in the shape of the phallus apex and furculae of males, with females of different species being virtually indistinguishable (Willemse 1971, 1972; Willemse et al. 2018). For this reason, identification to the species level is only possible by studying the male genitalia (Willemse et al. 2018). More recently, the discovery of some populations of *Oropodisma* that could not be confidently assigned to a particular species suggests the presence of further taxonomic diversity within the complex (Willemse and Willemse 2008).

Most species of the genus *Oropodisma* are exclusively distributed on a single mountain (*O.taygetosi* Willemse, 1972; *O.kyllinii* Willemse, 1971; *O.erymanthosi* Willemse, 1971; *O.lagrecai* Willemse, 1979; *O.tymphrestosi* Willemse, 1972) or nearby mountains (*O.parnassica*, *O.karavica* La Greca & Messina, 1977; *O.willemsei* La Greca & Messina, 1977), with estimated areas of occupancy of less than 50 km^2^ in most cases (Hochkirch et al. 2016). Only *O.chelmosi* Uvarov, 1942 and *O.macedonica* Ramme, 1951 have broader distributional ranges, with the former occurring in four mountain ranges in the Peloponnese Peninsula and the latter recorded in several mountains across central Greece, Albania, North Macedonia, and Kosovo (Willemse 1984; Cigliano et al. 2025). Their small distribution ranges, combined with the continuous decline of their populations due to climate warming, infrastructure development (e.g. wind power stations, ski centers), and cattle overgrazing, have led to the inclusion of all species in the IUCN Red List of Threatened Species under the Critically Endangered, Endangered, or Vulnerable categories (Hochkirch et al. 2016). Comparisons of historical records with recent resurveys of species distributions indicate that taxa found at the southernmost latitudes, particularly those inhabiting the Peloponnese Peninsula, have experienced significant shifts toward higher elevations and range contractions over the past 50 years, likely due to human-induced climate warming (Ortego 2025). The conservation value of all species within the genus *Oropodisma* underscores the necessity of reassessing their taxonomic status and identifying further hidden diversity, as this represents the most fundamental information required to guide future conservation actions (Hochkirch et al. 2016; e.g. Stefanidis et al. 2025).

In this study, we quantify morphological differences among taxa of the genus *Oropodisma* and reassess their taxonomic statuses. To this end, we first visited and obtained specimens from the type localities of all currently recognized taxa within the complex, as well as from two congeneric populations from central Greece that present an uncertain taxonomic status. Second, we employed a geometric morphometric approach to quantify differences among taxa based on the shape of male furculae and internal genitalia, the two traits that have been primarily used to describe the different species in previous studies (e.g. Willemse 1971, 1972; La Greca and Messina 1977; Willemse 1979). Our analyses confirmed the morphometric distinctiveness of most taxa and revealed the presence of two new species inhabiting Mounts Tzoumerka and Agrafa in the Pindus Range, central Greece. These findings contribute to increasing the known diversity of the genus *Oropodisma* and further highlight the key role of Mediterranean sky islands as hotspots of microgeographic speciation and local endemism (e.g. Peñas et al. 2005; Jiménez-Mejías et al. 2015; Ortego et al. 2024).

## ﻿Materials and methods

### ﻿Taxonomic sampling

During summers 2021 and 2023, we visited type localities of all described taxa within the genus *Oropodisma* (Cigliano et al. 2025) plus two congeneric populations from Mounts Tzoumerka and Agrafa that present an uncertain taxonomic status (Willemse and Willemse 2008; Table 1). We targeted to collect at least five male and five female adult individuals per population. However, the southernmost populations of the genus in the Peloponnese Peninsula have experienced marked population declines during past decades (Ortego 2025) and, after intensive prospecting during our two sampling campaigns, we could only find seven nymphs of *O.taygetosi*. For this reason, we requested a loan of five adult male museum specimens of this taxon, which are exclusively housed in the entomological collections of the Naturalis Biodiversity Center (**NNM**, Leiden, Netherlands). However, due to the very limited number of adult male specimens available in their collection, only two specimens were provided for invasive morphometric analyses. Spatial coordinates were recorded using a Global Positioning System (GPS) receiver. Whole specimens were preserved at −20 °C in 1500 µl of 100% ethanol until needed for morphometric analyses. Further details on sampled taxa and populations are presented in Table 1. Type specimens of newly described species have been deposited in Museo Nacional de Ciencias Naturales (**MNCN**, Madrid, Spain).

**Table 1.** Geographical location of topotype localities for species in the genus *Oropodisma* Uvarov, 1942 and type localities for the two newly described taxa.

Species	Code	*n*	Population	Country	Latitude, Longitude	Elevation
*Oropodismamacedonica* Ramme, 1951	MAC	5	Popova Shapka	North Macedonia	42.0228, 20.8708	1990
*Oropodismakaravica* La Greca & Messina, 1977	KAR	4	Mt. Karava	Greece	39.3280, 21.5722	1700
*Oropodismatymphrestosi* Willemse, 1972	TYM	5	Mt. Tymphristos	Greece	38.9390, 21.8077	1840
*Oropodismalagrecai* Willemse, 1979	LAG	5	Mt. Triandafillia	Greece	38.7031, 21.6866	1770
*Oropodismawillemsei* La Greca & Messina, 1977	WIL	5	Mt. Giona	Greece	38.5987, 22.2736	2120
*Oropodismaparnassica* (Scudder, 1897)	PAR	5	Mt. Parnassos	Greece	38.5308, 22.6197	2280
*Oropodismaerymanthosi* Willemse, 1971	ERY	5	Mt. Erymanthos	Greece	37.9516, 21.7938	1990
*Oropodismachelmosi* Uvarov, 1942	CHE	5	Mt. Chelmos	Greece	37.9755, 22.2028	2260
*Oropodismakyllinii* Willemse, 1971	KYL	5	Mt. Kyllini	Greece	37.9391, 22.3961	2350
*Oropodismataygetosi* Willemse, 1972	TAY	2*	Mt. Taygetus	Greece	36.9592, 22.3518	2260
*Oropodismatzoumerkae* sp. nov.	TZO	5	Mt. Tzoumerka	Greece	39.4031, 21.1632	1830
*Oropodismaagrafae* sp. nov.	AGR	5	Mt. Agrafa	Greece	39.1447, 21.6959	1780

*n* = number of male specimens examined; * Museum specimens from the entomological collections of the Naturalis Biodiversity Center (NNM, Leiden, Netherlands).

### ﻿Genitalia extraction and image acquisition

We examined five male specimens per taxon, except for *O.taygetosi* and *O.karavica*, for which only two and four specimens were available, respectively (Table 1). For the extraction of male genitalia, the abdominal tip of each specimen was removed and placed in a humid chamber for 48–72 hours to facilitate softening. The phallus apex was then carefully dissected using Swiss Rubis Tweezers (no. 5). Following dissection, genitalia were preserved in 99% glycerol for long-term storage in entomological collections. The phallus apex and furculae were photographed using a Leica Flexacam C3 camera mounted on a Zeiss Stemi 2000 stereomicroscope. Illustrations were generated using Adobe Photoshop CS6.

### ﻿Landmark digitizing

We used a geometric morphometrics approach to characterize differences in the shape of the phallus apex and furculae, which are diagnostic characters within the genus *Oropodisma* (Willemse 1971, 1972; Willemse et al. 2018). We digitized landmarks from each photograph using the r v. 4.2.3 (R Core Team 2021) package “*StereoMorph*” (Olsen and Westneat 2015). For furculae, we identified 10 homologous landmarks and eight curves, while for the phallus apex, we identified 16 landmarks and 12 curves (Fig. 2). Additionally, we used the r package “*geomorph*” (Adams and Otarola‐Castillo 2013) to sample 48 semilandmarks along the defined curves of the furculae and 64 semilandmarks for the phallus apex. All semilandmarks were converted to landmarks for subsequent analyses. We obtained shape files containing both raw and scaled coordinates, which were then used for downstream geometric morphometric analyses.

### ﻿Geometric morphometric analyses

To remove the effects of scale, rotation, and translation, all landmark and semilandmark configurations were subjected to Generalized Procrustes Analysis (GPA) using the r package “*geomorph*”. This step standardizes shape data, allowing comparisons solely based on shape variation rather than in differences in absolute size or orientation (Rohlf 1998). After standardizing shape data through GPA, we used the r package “*morpho*” (Schlager 2017) to perform Canonical Variate Analyses (CVA), which maximize shape differences among predefined groups (= taxa) while minimizing within-group variation. Based on the CVA, we calculated Mahalanobis distances to quantify morphological dissimilarity between each pair of taxa. To assess statistical significance, we conducted permutation tests (*n* = 10,000 permutations), evaluating whether differences between each pair of taxa were greater than expected by chance. We applied a false discovery rate (FDR) adjustment (5%, *q* < 0.05) to control for multiple tests.

## ﻿Results

### ﻿Taxonomy

#### ﻿ Oropodisma tzoumerkae

Taxon classificationAnimaliaOrthopteraAcrididae

Trillo & Ortego sp. nov.

3B9AA579-B525-549D-8E06-D1707190F6D5

https://zoobank.org/D91E26BE-3D2E-4C53-B367-E008148E6C2D

**Figure 1.**
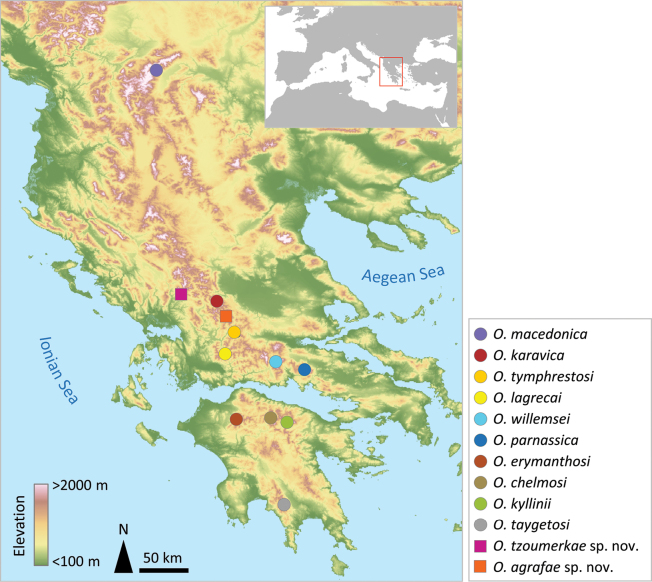
Map showing the geographical location of topotype localities for species in the genus *Oropodisma* Uvarov, 1942 and type localities for the two newly described taxa. Map in Plate Carrée projection.

Figs 1 , 3 , 5 , 6A , 7K , 8K

##### Diagnosis.

Differentiated from other species by the shape of the phallus apex (Fig. 7) and furculae (Fig. 8) of males. Phallus apex similar to *O.macedonica*, but presenting wider cingular valves, which lateral margins converge medially and become more pointed toward the tips (Fig. 7). Unlike *O.macedonica*, valves do not exhibit a basal swelling and remain straight or slightly concave at the base. Furculae of *O.tzoumerkae* present a wide base, similar to *O.karavica* and *O.lagrecai*, but are more elongated (Fig. 8). In comparison with *O.lagrecai*, furculae of *O.tzoumerkae* narrow more markedly medially, becoming slenderer (Fig. 8). Furculae of *O.karavica* show a very sharp angular bending, with a characteristic internal notch, whereas in *O.tzoumerkae* the curvature is more gradual and smooth (Fig. 8). Females indistinguishable from other species.

##### Description.

**Male**: small to medium-sized body (12.6–14.1 mm; Fig. 3A). Tegmina and wings absent. Head much shorter than pronotum, with frons slightly oblique. Fastigium sulcate. Eyes elliptical and obliquely flattened at the base. Vertical diameter of eyes approximately 1.1 times larger than the horizontal diameter. Eyes are dark brown to nearly black, sometimes with light-brown spots. Antennae stout, filiform, and short, similar to or slightly longer than the combined length of head and pronotum. The basal half of antennae is testaceous or yellowish, gradually darkening toward the tips. Pronotum short and broad, dorsally convex; anterior margin straight, posterior margin slightly convex. Pronotum with a densely punctured surface, lacking lateral carinae, and presenting continuous and deep transverse sulci. Prozona 1.8 times the length of the metazona. Prozona nearly cylindrical; metazona subcylindrical, slightly widening posteriorly. Median carina only visible and slightly raised in metazona. Dorsal surface of the pronotum black with a slight metallic sheen, similar to that on the top of the head and dorsal side of the abdomen. Lateral lobes of the pronotum with a characteristic whitish testaceous coloration, matching that of the thoracic sternum and the ventral parts of the head, including frons, genae, clypeus, labrum, mandibles and maxillae and labium. Abdomen slender, dorsally black with a distinctive bright yellowish median stripe; only the lower margins of mesonotum and metanotum bear a small testaceous stripe. Tympanal organ absent. Anterior and intermediate femora distinctly incrassate. Hind femora are robust, approximately 3.1 times as long as their maximum width, and present well-developed upper and lower carinae. Outer surface of the hind femur reddish to light brown; femora present two dark fasciae in the upper-inner area; inner surface testaceous with a blackish basal spot. Knees mostly black. Tibiae generally grayish-bluish. Last tergite bearing elongated furculae with a broad base, narrowing markedly medially, and tapering distally (as in Fig. 8K). Supra-anal plate triangular, with a deep median sulcus. Cerci small, narrowly triangular and shorter than the supra-anal plate. Phallus apex with narrow cingular valves, similar in shape to the valves (as in Fig. 7K); valves nearly equal in width and length to the cingular valves; lateral margins of the cingular valves converge medially and become markedly pointed toward the tips; lateral margins of the valves straight or slightly concave, lacking a basal swelling (as in Fig. 7K). **Female**: small to medium-sized body (17.0–19.3 mm), larger and more robust than males (Fig. 3B). Pronotum broader and more trapezoidal than in males. Pronotum presents continuous transverse sulci, but these are less deeply impressed than in males. Antennae much shorter than the combined length of the head and pronotum. Posterior margin of the pronotum bordered with alternating light testaceous and dark stripes. Abdomen considerably stouter than in males. Anterior and intermediate femora not or only slightly incrassate. Supra-anal plate triangular, with short, conical cerci. Ovopositor valves robust, strongly sinuate, with a curved, pointed apex. Lower valve with two acute lateral teeth. Other characteristics resemble the male.

##### Type material.

***Holotype***: • 1 ♂, Greece, Epirus, Mount Tzoumerka, 10 August 2023, J. Ortego & J. Gutiérrez-Rodríguez leg.; deposited in Museo Nacional de Ciencias Naturales (MNCN, Madrid, Spain; accession number: MNCN_Ent 429979) (Fig. 3A). ***Paratypes***: • 5 ♂ and 6 ♀, same data as holotype; deposited in Estación Biológica de Doñana (CSIC-EBD, Seville, Spain; accession numbers: 5 ♂, JO-15944, JO-15945, JO-15946, JO-15947, JO-15949 & 5 ♀, JO-15954, JO-15955, JO-15956, JO-15957, JO-15958) and Museo Nacional de Ciencias Naturales (MNCN, Madrid, Spain; accession number: 1 ♀, MNCN_Ent 429980) (Fig. 3B).

##### Type locality.

Greece, Epirus, Arta, Athamanio, Mount Tzoumerka, 39.4031°N, 21.1632°E, 1830 m.a.s.l., 10 August 2023; open alpine habitat characterized by stony ground and sparse plant cover (Fig. 6A).

##### Male measurements

(mm) (*n* = 5). Body length: 12.6–14.1 (mean = 13.4); pronotum length: 2.7–3.0 (mean = 2.8); hind femur length: 6.9–7.4 (mean = 7.2); furcula length: 3.9–4.7 (mean = 4.4); furcula basal width: 2.1–2.8 (mean = 2.5).

##### Female measurements

(mm) (*n* = 5). Body length: 17.0–19.3 (mean = 18.2); pronotum length: 3.3–3.7 (mean = 3.5 cm); hind femur length: 8.5–9.3 (mean = 8.9).

##### Habitat.

Open alpine habitat characterized by stony ground, moderate slopes, and sparse plant cover with scattered patches of evergreen scrubs (*Juniperus* sp., *Daphne* sp.) (Fig. 6A). Similar habitat as previously described for other species of *Oropodisma*.

##### Distribution.

Known only from the type locality (Fig. 1).

**Figure 2.**
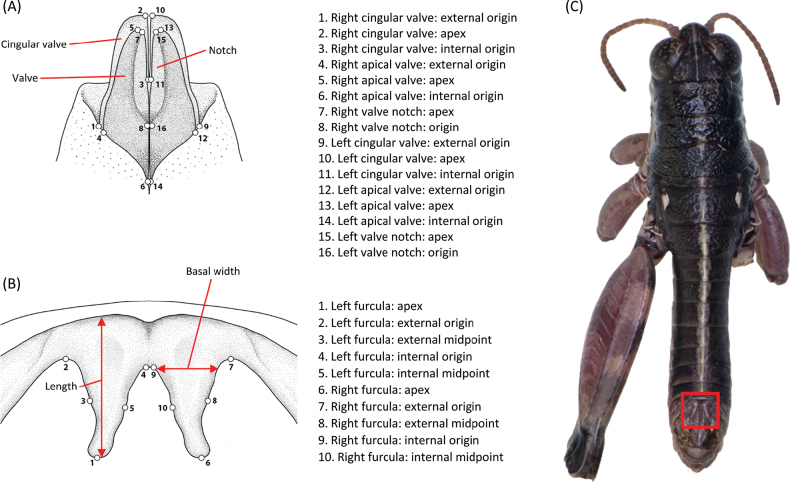
Landmarks used to characterize structures in males of *Oropodisma* species **A** phallus apex **B** furculae **C** shows a male with a red square indicating the location of furculae. Drawings and photograph: M. Trillo.

**Figure 3.**
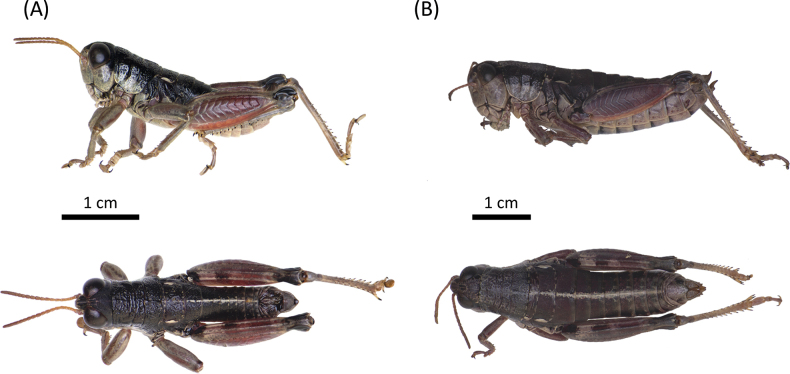
Habitus of *Oropodismatzoumerkae* sp. nov., in lateral and dorsal view **A** male (holotype, MNCN) **B** female (paratype, MNCN). Photographs: M. Trillo.

##### Etymology.

A toponimic name. The name *tzoumerkae* refers to Mount Tzoumerka (Pindus range, Greece), the area where the species was found.

##### Suggested common name.

Tzoumerka mountain grasshopper.

#### ﻿ Oropodisma agrafae

Taxon classificationAnimaliaOrthopteraAcrididae

Trillo & Ortego sp. nov.

FEF7392C-1477-5EE5-97B4-FF6F48A43C8D

https://zoobank.org/21426509-A078-49D3-BFD7-0D6AD8A3E8D5

**Figure 4.**
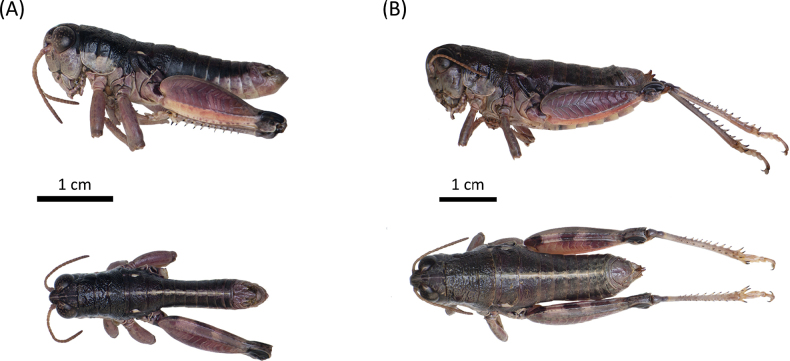
Habitus of *Oropodismaagrafae* sp. nov. in lateral and dorsal view **A** male (holotype, MNCN) **B** female (paratype, MNCN). Photographs: M. Trillo.

**Figure 5.**
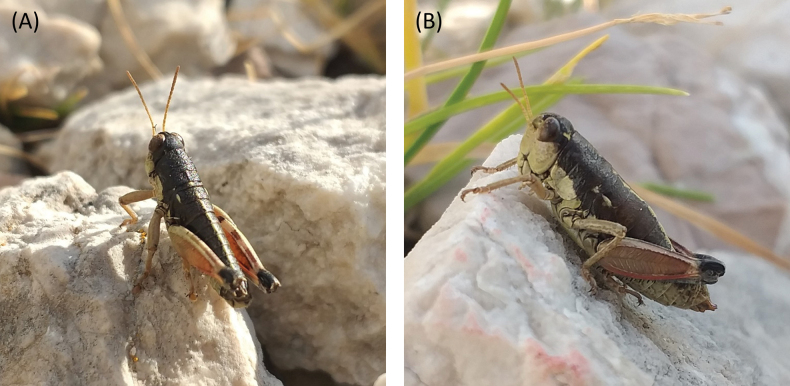
*Oropodismatzoumerkae* sp. nov. photographed at Mt. Tzoumerka, Greece, 10 August 2023 **A** male **B** female. Photographs: J. Ortego.

**Figure 6.**
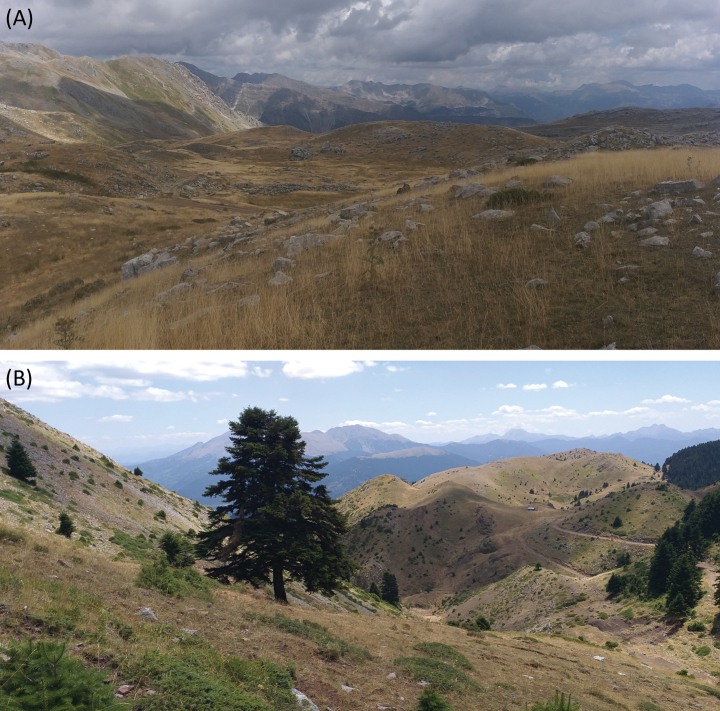
Habitat of the new *Oropodisma* species **A***O.tzoumerkae* sp. nov., Mount Tzoumerka (10 August 2023) **B***O.agrafae* sp. nov., Mount Agrafa (08 August 2023). Photographs: J. Ortego.

**Figure 7.**
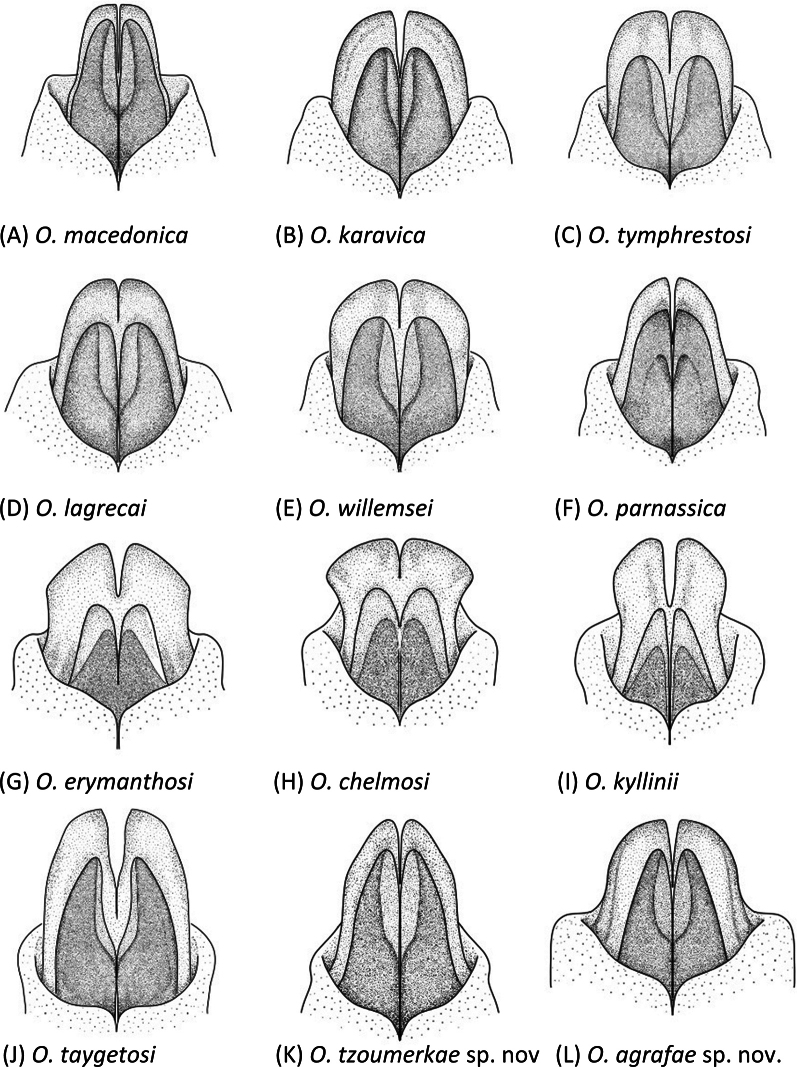
Dorsal view of the phallus apex of males in *Oropodisma* species. Drawings: M. Trillo.

**Figure 8.**
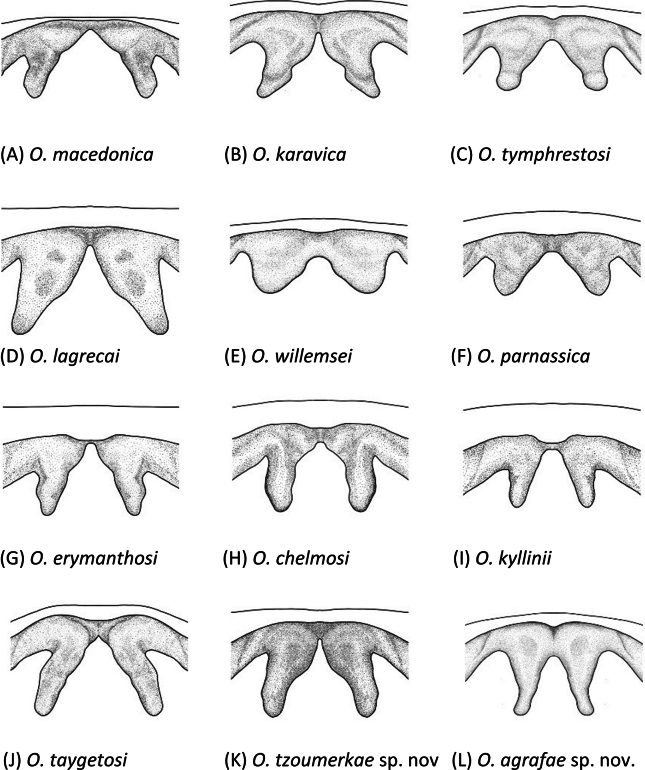
Dorsal view of furculae of males in *Oropodisma* species. Drawings: M. Trillo.

Figs 1 , 4 , 5 , 6B , 7L , 8L

##### Diagnosis.

Differentiated from other species by the shape of the phallus apex (Fig. 7) and furculae (Fig. 8) of males. Phallus apex similar to *O.karavica*, but cingular valves are slightly wider basally and valves are more pointed apically and have wider notches (Fig. 7). Furculae of *O.agrafae* are distinctively slender and elongated (Fig. 8). Furculae wide at the base with lateral margins angling inward, tapering distally and redirecting outward near the apex, which is slightly lobulated (Fig. 8). Females indistinguishable from other species.

##### Description.

**Male**: small to medium-sized body (15.7–16.7 mm; Fig. 4A). Tegmina and wings absent. Head much shorter than pronotum, with frons slightly oblique. Fastigium sulcate. Eyes elliptical and obliquely flattened at the base. Vertical diameter of eyes approximately 1.1 times larger than the horizontal diameter. Eyes dark brown to nearly black, presenting conspicuous light brown spots. Antennae stout, filiform, and short, similar to or slightly longer than the combined length of head and pronotum. The basal half of antennae is testaceous or yellowish, gradually darkening toward the tips. Pronotum short and broad, dorsally convex; anterior margin straight, posterior margin slightly convex. Pronotum with a densely punctured surface, lacking lateral carinae, and presenting continuous and deep transverse sulci. Prozona 1.8 times the length of the metazona. Prozona nearly cylindrical; metazona subcylindrical, slightly widening posteriorly. Median carina only visible and slightly raised in metazona. Dorsal surface of the pronotum black with a slight metallic sheen, similar to that on the top of the head and dorsal side of the abdomen. Lateral lobes of the pronotum with a characteristic whitish testaceous coloration, matching that of the thoracic sternum and the ventral parts of the head, including frons, genae, clypeus, labrum, mandibles and maxillae and labium. Abdomen slender, dorsally black with a distinctive bright yellowish median stripe. Only the lower margins of mesonotum and metanotum bear a small testaceous stripe. Tympanal organ absent. Anterior and intermediate femora distinctly incrassate. Hind femora are robust, approximately 3.1 times as long as their maximum width, and present well-developed upper and lower carinae. Outer surface of the hind femur reddish to light brown; femora present two dark fasciae in the upper-inner area; inner surface testaceous with a blackish basal spot. Knees mostly black. Tibiae generally grayish-bluish. Last tergite bearing distinctly slender and elongated furculae (as in Fig. 8I). Furculae broad at the base, with lateral margins angling inward, tapering distally, and curving outward near the apex, which is slightly lobulated. Supra-anal plate triangular, with a deep median sulcus (as in Fig. 8I). Cerci small, narrowly triangular and shorter than the supra-anal plate. Phallus apex with rounded cingular valves distinctly wider and longer than the valves (as in Fig. 7I). Valves taper apically, with straight lateral margins, a pointed apex, and broad internal notches (as in Fig. 7I). **Female**: small to medium-sized body (18.3–20.6 mm), larger and more robust than males (Fig. 4B). Pronotum broader and more trapezoidal than in males. Pronotum presents continuous transverse sulci, but these are less deeply impressed than in males. Antennae much shorter than the combined length of the head and pronotum. Posterior margin of the pronotum bordered with alternating light testaceous and dark stripes. Abdomen considerably stouter than in males. Anterior and intermediate femora not or only slightly incrassate. Supra-anal plate triangular, with short, conical cerci. Ovopositor valves robust, strongly sinuate, with a curved, pointed apex. Lower valve with two acute lateral teeth. Other characteristics resemble the male.

##### Type material.

***Holotype***: • 1 ♂, Greece, Central Greece, Mount Agrafa, 08 August 2023, J. Ortego & J. Gutiérrez-Rodríguez leg.; deposited in Museo Nacional de Ciencias Naturales (MNCN, Madrid, Spain; accession number: MNCN_Ent 429977) (Fig. 4A). ***Paratypes***: • 5 ♂ and 6 ♀, same data as holotype; deposited in Estación Biológica de Doñana (CSIC-EBD, Seville, Spain; accession numbers: 5 ♂, JO-15731, JO-15732, JO-15733, JO-15734, JO-15736 & 5 ♀, JO-15741, JO-15742, JO-15745, JO-17560, JO-17561) and Museo Nacional de Ciencias Naturales (MNCN, Madrid, Spain; accession number: 1 ♀, MNCN_Ent 429978) (Fig. 4B).

##### Type locality.

Greece, Central Greece, Evrytania, Kamaria, Mount Agrafa, 39.1447°N, 21.6959°E, 1780 m.a.s.l., 08 August 2023; open alpine habitat characterized by stony ground and sparse plant cover (Fig. 6B).

##### Male measurements

(mm) (*n* = 5). Body length: 15.7–16.7 (mean = 16.2); pronotum length: 3.2–3.5 (mean = 3.4); hind femur length: 7.9–8.4 (mean = 8.2); furcula length: 5.9–7.0 (mean = 6.6); furcula basal width: 3.3–3.9 (mean = 3.7).

##### Female measurements

(mm) (*n* = 5). Body length: 18.3–20.6 (mean = 19.3); pronotum length: 3.8–4.0 (mean = 3.9); hind femur length: 9.6–10.9 (mean = 10.2).

##### Habitat.

Open alpine habitat characterized by stony ground, moderate slopes, and sparse plant cover with scattered patches of evergreen scrubs (*Juniperus* sp.) (Fig. 6B). Similar habitat as previously described for other species of *Oropodisma*.

##### Distribution.

Known only from the type locality (Fig. 1).

##### Etymology.

A toponimic name. The name *agrafae* refers to Mount Tzoumerka (Pindus range, Greece), the area where the species was found.

##### Suggested common name.

Agrafa mountain grasshopper.

### ﻿Key to *Oropodisma* species

**Table:** 

1	Cingular valves wide and medially constricted, with distinctly concave lateral margins (as in Fig. 7G–I)	**2**
–	Cingular valves not medially constricted (as in Fig. 7A, F, J–L)	**4**
2	Cingular valves markedly constricted medially, with distinctly concave lateral margins; the two apical lobes of the cingular valves are broad, quadrangular, and narrowly incised; the incision separating the two lobes of the cingular valves does not reach the tip of the valves (as in Fig. 7H); only distributed on Mounts Panachaiko, Chelmos, Maenalon and Parnon (Greece)	***O.chelmosi***
–	Cingular valves only slightly constricted medially; the two apical lobes of the cingular valves are separated by a wide incision that reaches or nearly reaches the tips of the valves (as in Fig. 7G, I)	**3**
3	Apical lobes of the cingular valves as long as or longer than their basal width; tips of the valves are markedly pointed (as in Fig. 7I); only distributed on Mount Kyllini (Greece)	***O.kyllinii***
–	Cingular valves markedly wide; apical lobes of the cingular valves trapezoidal and shorter than their basal width; the tips of the valves are rounded (as in Fig. 7G); only distributed on Mount Erymanthos (Greece)	***O.erymanthosi***
4	Furculae short and broadly rounded; furculae length equal to or shorter than their basal width (as in Fig. 8E, F)	**5**
–	Furculae elongated, longer than their basal width (as in Fig. 8A–D, J–L)	**6**
5	Cingular valves gradually narrowing toward the tips; internal and lateral margins of the valves are straight and rounded, respectively; medial internal area of the valves with a small bell-shaped notch (as in Fig. 7F); only distributed on Mounts Parnassos and Elikonas (Greece)	***O.parnassica***
–	Cingular valves widening medially, with rounded apical lobes; valves gradually narrowing toward the tips, which are rounded; the valves have a wide internal notch extending from the medial area almost to the tip (as in Fig. 7E); distributed on Mount Giona and on nearby mountains (Greece)	***O.willemsei***
6	Cingular valves narrow, similar in shape to the valves; valves nearly as wide and long as the cingular valves (as in Fig. 7A, K)	**7**
–	Cingular valves distinctly wider and longer than the valves (as in Fig. 7B–D, L)	**8**
7	Lateral margins of the cingular valves converge medially and become markedly pointed at the tips; valves do not exhibit a basal swelling and remain straight or slightly concave (as in Fig. 7K); only distributed on Mount Tzoumerka (Greece)	***O.tzoumerkae* sp. nov.**
–	Cingular valves markedly narrow, nearly equal in size to the valves; valves with a basal swelling and slightly convex at the base; the tips of the lobes of the cingular valves are markedly flat (as in Fig. 7A); distributed in Kosovo, Albania, North Macedonia, and the northeastern part of the Pindus range in Greece	***O.macedonica***
8	Furculae markedly slender and elongated (as in Fig. 8J, L)	**9**
–	Furculae not slender (as in Fig. 8B–D)	**10**
9	Cingular valves broad, apically elongated and much larger than the valves; valves pointed, tapering apically, with convex lateral margins and narrow internal notches (as in Fig. 7J); furculae do not widen at the base, with lateral margins angling outward (as in Fig. 8J); only distributed on Mount Taygetus (Greece)	***O.taygetosi***
–	Cingular valves broad, apically rounded and not elongated; valves pointed, tapering apically, with straight lateral margins and wide internal notches (as in Fig. 7L); furculae wide at the base with lateral margins angling inward (as in Fig. 8L); only distributed on Mount Agrafa (Greece)	***O.agrafae* sp. nov.**
10	Furculae presenting a very sharply angularly bending, with a characteristic internal notch (as in Fig. 8B); only present on Mount Karava and nearby mountains (Greece)	***O.karavica***
–	Furculae without an internal notch (as in Fig. 8C-D)	**11**
11	Furculae large, longer than their basal width (as in Fig. 8D); cingular valves gradually narrowing from the base to the tips (as in Fig. 7D); only distributed on Mount Triandafillia (Greece)	***O.lagrecai***
–	Furculae as long as or shorter than their basal width (as in Fig. 8C); cingular valves broadly rounded, forming wide lobes; only distributed on Mount Tymphristos and on nearby mountains	***O.tymphrestosi***

### ﻿Geometric morphometric analyses

**Figure 9.**
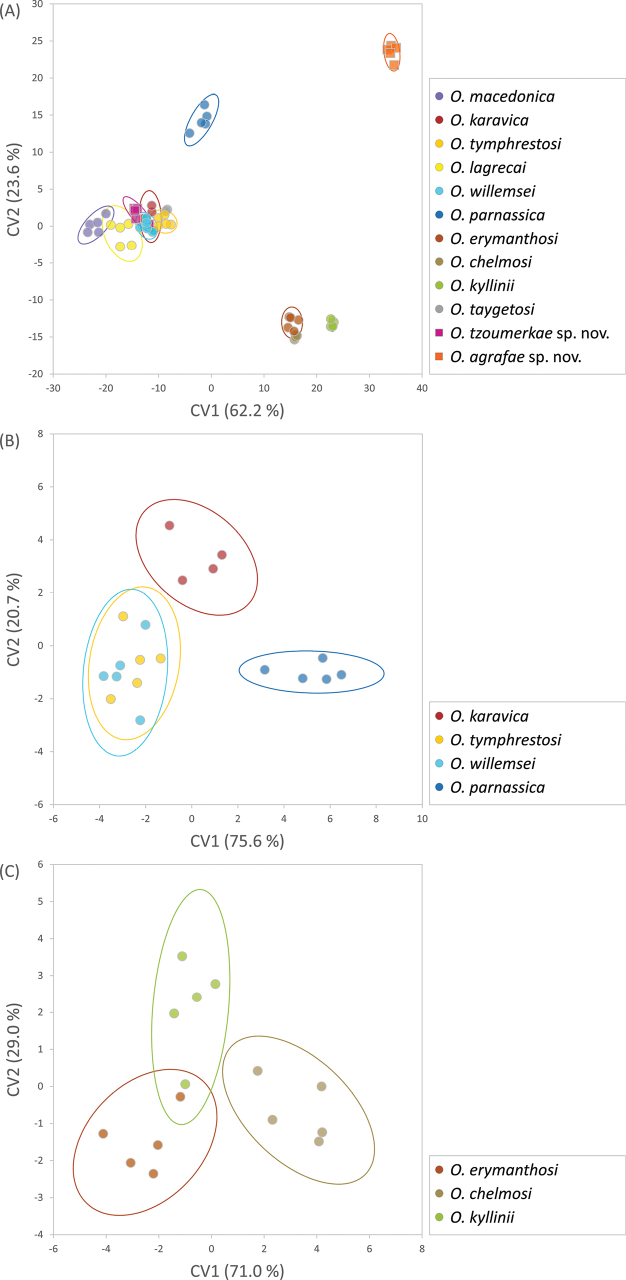
Canonical Variate Analyses (CVA) comparing landmark coordinates of the phallus apex among the different species of *Oropodisma*. Analyses were performed hierarchically, (**A**) first including all taxa and (**B, C**) then analyzing separately two groups of taxa that were not significantly different in either of the two studied traits (the phallus apex and furculae) in analyses including all taxa (Table 2A). Ellipses represent 95% confidence intervals.

**Figure 10.**
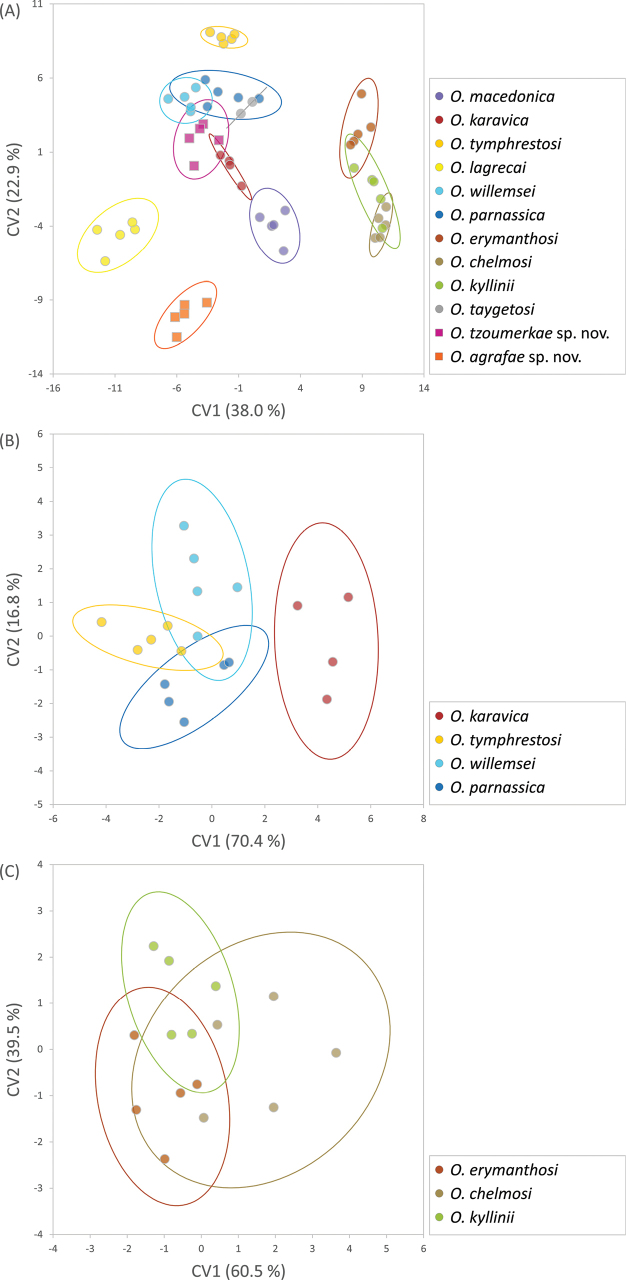
Canonical Variate Analyses (CVA) comparing landmark coordinates of male furculae among the different species of *Oropodisma*. Analyses were performed hierarchically, (**A**) first including all taxa and (**B, C**) then analyzing separately two groups of taxa that were not significantly different in either of the two studied traits (the phallus apex and furculae) in analyses including all taxa (Table 2A). Ellipses represent 95% confidence intervals.

**Figure 11.**
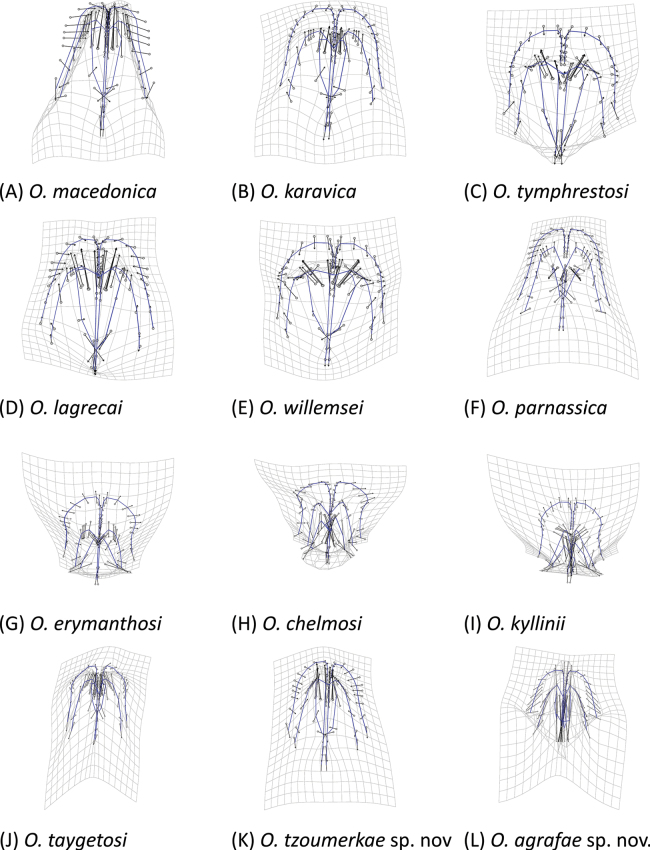
Deformation grids and vectorized shapes of the phallus apex of males of *Oropodisma* species.

**Figure 12.**
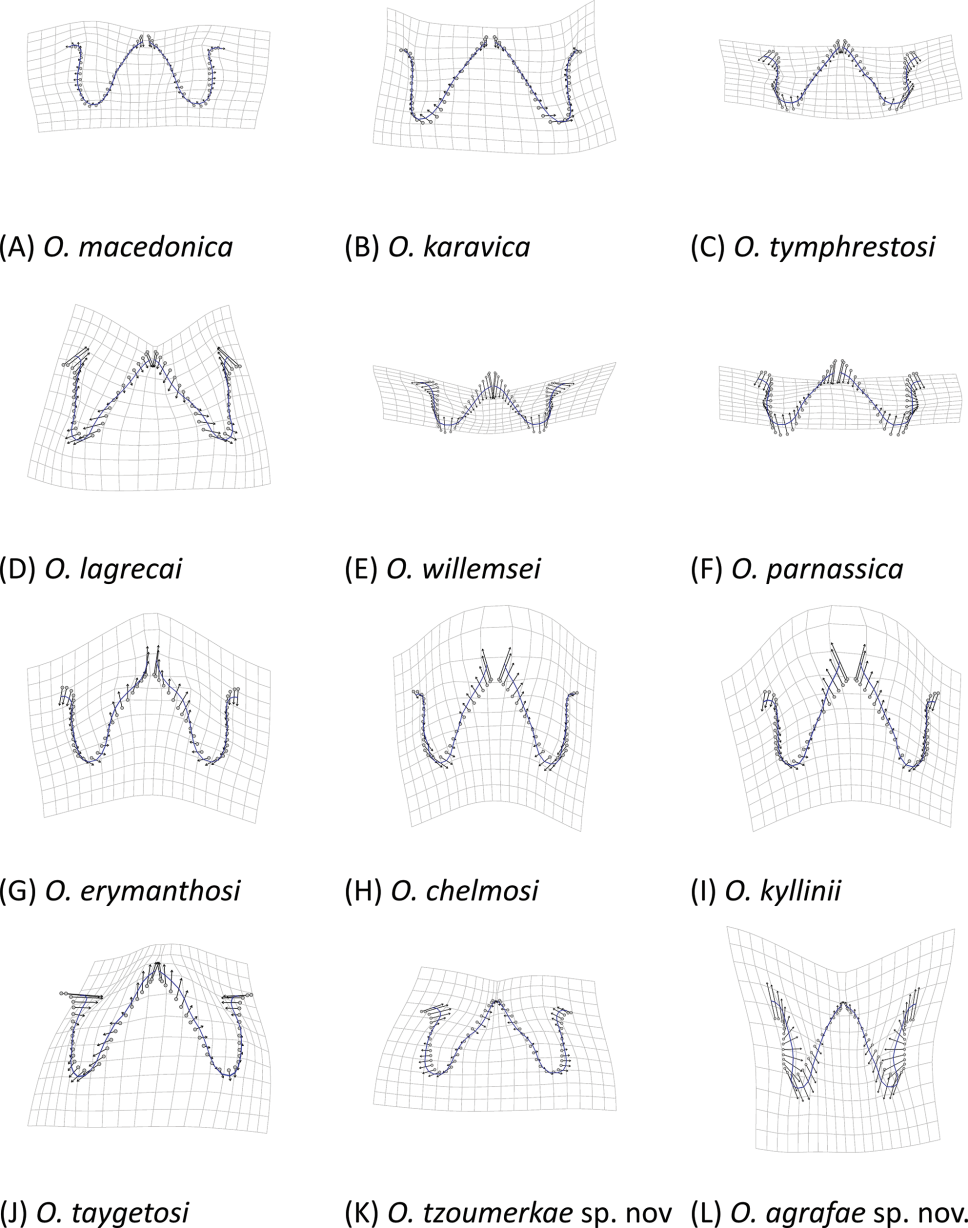
Deformation grids and vectorized shapes of furculae of males of *Oropodisma* species.

Geometric morphometric analyses of the phallus apex and furculae are presented in Table 2 and Figs 9–12. A Canonical Variate Analysis (CVA) of the phallus apex revealed that the different taxa grouped into four main clusters: one consisting exclusively of *O.agrafae* sp. nov., one including only *O.parnassica*, another comprising the three taxa distributed in the northern Peloponnese Peninsula (*O.erymanthosi*, *O.chelmosi*, and *O.kyllinii*), and a final cluster containing the remaining taxa (*O.macedonica*, *O.karavica*, *O.tymphrestosi*, *O.lagrecai*, *O.willemsei*, *O.taygetosi*, and *O.tzoumerkae* sp. nov.) (Fig. 9). Mahalanobis distances were significantly different among species from different clusters but not among taxa within each of these four main groups (Table 2A). The only exception was *O.parnassica*, which, despite forming its own cluster, was not significantly differentiated from *O.karavica*, *O.tymphrestosi*, *O.lagrecai*, *O.willemsei*, and *O.tzoumerkae* sp. nov. (Table 2A). A CVA of male furculae similarly identified four main clusters: one including only *O.agrafae* sp. nov., one consisting solely of *O.lagrecai*, another grouping the three taxa from the northern Peloponnese Peninsula (*O.erymanthosi*, *O.chelmosi*, and *O.kyllinii*), and a final cluster encompassing the remaining taxa (*O.macedonica*, *O.karavica*, *O.tymphrestosi*, *O.willemsei*, *O.parnassica*, *O.taygetosi*, and *O.tzoumerkae* sp. nov.) (Fig. 10). Mahalanobis distances for male furculae were significantly different in most pairwise comparisons, except among the three northern Peloponnese taxa (*O.erymanthosi*, *O.chelmosi*, and *O.kyllinii*) and four taxa from central Greece (*O.karavica*, *O.tymphrestosi*, *O.willemsei*, and *O.parnassica*) (Table 2A). Notably, *O.agrafae* sp. nov. was significantly differentiated from all other taxa based on both the phallus apex and furculae, while *O.tzoumerkae* sp. nov. was significantly differentiated from all other taxa based on furculae (Table 2A; Figs 9, 10). The four taxa from central Greece (*O.karavica*, *O.tymphrestosi*, *O.willemsei*, and *O.parnassica*) and the three from the northern Peloponnese (*O.erymanthosi*, *O.chelmosi*, and *O.kyllinii*) did not exhibit significant differences in either of the two studied traits (Table 2A). To further investigate potential morphological differences within these two groups, we conducted separate CVAs. These analyses revealed that most pairs of taxa within each group were significantly differentiated in at least one of the two studied traits (Table 2B; Figs 9B, C, 10B, C). The only exception was the species pair *O.tymphrestosi* and *O.willemsei*, which did not show statistically significant differences in the shape of either the phallus apex or furculae (Table 2B; Figs 9B, 10B).

**Table 2.** Mahalanobis distances (*D*) between the different species of *Oropodisma* obtained through a Canonical Variates Analysis (CVA) for the phallus apex (above the diagonals) and furculae (below the diagonals) of males. Analyses were performed hierarchically, (A) first including all taxa and (B) then analyzing separately two groups of taxa (KAR-TYM-WIL-PAR and ERY-CHE-KYL) that were not significantly different in either of the two studied traits (the phallus apex and furculae) in analyses including all taxa (see Table 2A). Values in bold indicate statistically significant Mahalanobis distances after false discovery rate adjustment (FDR) to control for multiple tests (FDR of 5%, *q* < 0.05). Due to small sample sizes (*n* = 2; see Table 1) for *O.taygetosi*, statistical significance of pairwise comparisons involving this taxon were not calculated. Species codes as described in Table 1.

**(A) All taxa**												
**Code**	**MAC**	**KAR**	**TYM**	**LAG**	**WIL**	**PAR**	**ERY**	**CHE**	**KYL**	**TAY**	**TZO**	**AGR**
MAC	–	14.163	18.831	11.281	16.324	**28.473**	**41.936**	**42.101**	**48.346**	24.011	16.050	**61.264**
KAR	**12.491**	–	10.346	10.756	11.118	20.892	**31.933**	**33.556**	**39.623**	15.934	11.480	**51.308**
TYM	**16.130**	10.846	–	12.829	9.519	18.995	**29.377**	**32.181**	**36.953**	18.726	12.482	**49.869**
LAG	**17.017**	**15.543**	**19.685**	–	11.715	26.369	**36.142**	**37.729**	**43.626**	21.231	14.845	**57.007**
WIL	**14.024**	9.846	7.742	**16.485**	–	22.687	**32.396**	**34.777**	**39.608**	19.728	12.062	**52.759**
PAR	**12.888**	8.262	6.656	**17.667**	6.210	–	**35.107**	**36.982**	**38.599**	22.902	21.165	**40.118**
ERY	**13.929**	**13.878**	**15.621**	**22.139**	**17.154**	**14.325**	–	15.908	16.515	33.426	**34.624**	**42.302**
CHE	**11.769**	**15.270**	**19.115**	**22.791**	**19.278**	**16.789**	10.793	–	18.184	35.036	**37.260**	**43.693**
KYL	**12.820**	**13.816**	**16.648**	**22.789**	**17.894**	**15.246**	10.318	7.349	–	39.294	**40.885**	**40.392**
TAY	14.411	16.228	15.869	16.759	17.023	15.664	15.306	17.596	18.252	–	15.281	50.817
TZO	**13.325**	**14.012**	**14.257**	**12.639**	**14.179**	**13.418**	**16.551**	**17.886**	**17.959**	9.131	–	**54.020**
AGR	**14.983**	**13.110**	**19.749**	**17.309**	**15.866**	**16.347**	**21.876**	**19.697**	**19.400**	23.466	**19.637**	–
**(B) Groups of taxa**												
**Code**	**KAR**	**TYM**	**WIL**	**PAR**	**ERY**	**CHE**	**KYL**					
KAR	–	4.968	**5.413**	**6.603**								
TYM	**6.780**	–	2.071	**7.770**								
WIL	**5.253**	3.452	–	**8.140**								
PAR	**5.442**	3.288	3.203	–								
ERY					–	**5.893**	**4.048**					
CHE					**2.770**	–	**4.953**					
KYL					2.302	2.619	–					

## ﻿Discussion

In this study, we applied a geometric morphometric approach to quantify morphological differences among taxa of the genus *Oropodisma*, supporting the distinctiveness of most currently recognized taxa and revealing the presence of two new species: *Oropodismatzoumerkae* sp. nov. and *Oropodismaagrafae* sp. nov. These two taxa, named after the mountain ranges they inhabit, increase the number of species within the genus *Oropodisma* to twelve (Table 1; Cigliano et al. 2025). The populations of the new taxa were first discovered by Willemse and Willemse (2008), but could not be unambiguously assigned to any previously described species. They noted that males from the Mt. Agrafa population exhibited particularly long and slender furculae, suggesting that they might represent an undescribed species. Our detailed analyses corroborated not only the distinct male furculae of the Mount Agrafa population, but also the very distinctive shape of the phallus apex. In fact, geometric morphometric analyses revealed that *O.agrafae* sp. nov. is particularly well differentiated from the other species in the multivariate space, making it one of the most distinctive *Oropodisma* taxa based on the two studied traits (Table 2A; Figs 9A, 10A). Although *O.tzoumerkae* sp. nov. is comparatively more similar to other taxa inhabiting nearby mountain ranges, its distinctive furculae also support its species-level status. Conversely, geometric morphometric analyses indicate that the taxa *O.tymphrestosi* and *O.willemsei*, distributed in adjacent mountain ranges of central Greece, do not show statistically significant differences in either of the two studied traits. While this finding raises doubts about their taxonomic status, it might also reflect certain limitations of geometric morphometric approaches in capturing subtle differences in the complex, three-dimensional structures of internal male genitalia. For this reason, we prefer not to take a taxonomic decision—namely, the synonymization of *O.willemsei* with *O.tymphrestosi*—until additional evidence from ongoing genomic-based studies becomes available.

In line with previous taxonomic studies on the genus (Uvarov 1942; Willemse 1971, 1972), our geometric morphometric analyses supported the similarity among some species from central Greece (particularly *O.karavica*, *O.tymphrestosi*, *O.willemsei*, and *O.parnassica*), as well as among the three species inhabiting the mountains from the northern Peloponnese Peninsula (*O.erymanthosi*, *O.chelmosi*, and *O.kyllinii*). Phenotypic affinities among taxa distributed in adjacent mountain ranges likely reflect the predominant role of geography in speciation within this complex, consistent with genetic-based biogeographic inferences from other recent radiations of flightless alpine grasshoppers (Ortego and Knowles 2022) and bush crickets (Ortego et al. 2024). As observed in other alpine organisms from temperate regions, populations of *Oropodisma* likely formed more continuous populations during glacial periods, becoming small and highly fragmented during warm interglacials (Bennett and Provan 2008; Stewart et al. 2010; e.g. Tonzo and Ortego 2021; Ortego and Knowles 2022). Consistent with findings for most complexes of melanopline grasshoppers (e.g. Huang et al. 2020; Ortego and Knowles 2022; Hill 2024), *Oropodisma* likely radiated during the Pleistocene (<2.6 Ma), probably during short interglacial periods when populations became fragmented and geographically isolated on mountain tops (i.e. interglacial refugia; Stewart et al. 2010). Under this scenario of interglacial speciation, and considering the niche conservatism and allopatric distributions of all taxa, divergence in male genital structures driven by genetic drift may have played a key role in the rapid evolution of reproductive isolation and speciation in the complex (e.g. Huang et al. 2020). The presence of pre- and postzygotic barriers to gene flow among the described taxa remains to be experimentally tested (e.g. Coyne and Orr 1989; Hoskin et al. 2005; Saldamando et al. 2005). Laboratory mating and breeding attempts between taxa with similar morphologies and inhabiting adjacent mountain ranges (e.g. *O.tymphrestosi* and *O.willemsei*) could help elucidate the taxonomic significance of observed subtle differences in male genitalia and their role in reproductive isolation (i.e. mating success and offspring viability) and speciation in both *Oropodisma* and other melanopline radiations (Huang et al. 2020; Ortego and Knowles 2022; Hill 2024).

Collectively, our study supports the taxonomic distinctiveness of most *Oropodisma* taxa, and the description of two additional species anticipates further discovery of microendemic cryptic species in the Mediterranean biodiversity hotspot. The small distribution ranges of most taxa and documented population declines over the past 50 years (Ortego 2025) indicate their high vulnerability to climate change and other environmental disturbances, underscoring the need to assess the conservation status of the two new species and re-evaluate all others (Hochkirch et al. 2016; Ortego 2025). Integrating traditional taxonomic knowledge with genomic data for species delimitation (Sukumaran et al. 2021; e.g. Noguerales et al. 2018; Tonzo et al. 2019) and biogeographic inference (e.g. Schoville et al. 2012; Huang et al. 2020) may help clarify the taxonomic status of certain populations and shed light on the timing and proximate processes underlying the formation of narrowly distributed species of high conservation value (Hochkirch et al. 2016).

## Supplementary Material

XML Treatment for Oropodisma tzoumerkae

XML Treatment for Oropodisma agrafae

## ﻿Additional information

### Author ORCIDs

Marina Trillo https://orcid.org/0000-0002-3650-138X

Joaquín Ortego https://orcid.org/0000-0003-2709-429X

### Data availability

Dataset of linear measurements and images and input files for geometric morphometric analyses are available for download from Figshare, https://doi.org/10.6084/m9.figshare.28413104.
